# From single errors to negative psychological momentum in youth basketball: post-error mechanisms and coach-mediated resetting

**DOI:** 10.3389/fpsyg.2026.1961600

**Published:** 2026-09-23

**Authors:** ZhengYu Shi, Yifu Zhu, Xitang Zhao

**Affiliations:** 1 Zhumadian Vocational and Technical College, Zhumadian, Henan, China; 2 Hubei Urban Construction Vocational and Technical College, Wuhan, Hubei, China; 3 Nanjing Sport Institute, Nanjing, Jiangsu, China

**Keywords:** attentional control, coach feedback, fear of failure, performance errors, psychological momentum, youth basketball

## Abstract

Errors in youth basketball are often treated as isolated technical events; however, their psychological effects may persist across subsequent possessions. This Mini Review integrates evidence on psychological momentum, post-error affect, attentional control, efficacy-related beliefs, social evaluation, and coach feedback to explain how a single error may develop into repeated performance failure. A focused narrative search prioritized evidence from youth basketball, followed by basketball in general, youth team sports, and broader sport psychology when direct evidence was limited. The review proposes a self-reinforcing error–emotion–attention–efficacy–error cycle, in which an initial error may leave residual negative affect, shift attention toward self-focused or evaluative information, reduce perceived efficacy and control, and impair subsequent decision-making or execution. In youth athletes, coach and peer evaluation and fear of failure may amplify this process by increasing the social evaluative meaning of errors. Conversely, supportive and task-focused coach feedback may facilitate emotional resetting, attentional refocusing, and efficacy restoration, whereas controlling feedback may prolong negative momentum. The complete pathway remains an integrative hypothesis rather than an established causal model. Future possession-level research is needed to test these mechanisms directly in youth basketball.

## 1 Introduction

Basketball requires rapid transitions, continuous decision-making, and immediate responses to performance outcomes. A turnover, poor shot selection, or defensive error may therefore affect not only the current possession but also subsequent play. Studies have identified short-term scoring momentum in basketball, while time-out effects appear to vary with game context rather than being uniformly beneficial (Sampaio et al., 2013; Qiu et al., 2024, 2025; Carta et al., 2026). However, these performance fluctuations should not be equated directly with athletes’ subjective psychological momentum.

Psychological momentum (PM) is a dynamic, history-dependent process in which prior competitive events influence subsequent cognitive, affective, motivational, and behavioral states (Gernigon et al., 2010). Evidence suggests that PM can build across sequences of success or failure, operate across interconnected timescales, and be altered by interruptions such as timeouts (Den Hartigh et al., 2016; Briki and Markman, 2018; Den Hartigh and Gernigon, 2018). Proposed mechanisms include changes in efficacy, motivation, and concentration rather than outcome streaks alone (Morgulev, 2023).

In this review, negative psychological momentum refers specifically to a history-dependent, cross-possession process in which an adverse event is followed by psychological changes that persist into subsequent possessions and bias later states or behaviors in a self-reinforcing direction. Accordingly, post-error anxiety, rumination, attentional disruption, and reduced self-efficacy are treated as candidate components or mechanisms of negative PM rather than as negative PM itself, whereas temporary performance deterioration is treated as a possible behavioral consequence rather than sufficient evidence of a momentum state. The defining feature is therefore the temporal carryover across possessions, rather than the presence of any single negative response or a short sequence of poor outcomes.

This process may be particularly relevant in youth team sports, where errors occur within a socially evaluative environment. Real-time evidence from high school volleyball shows that self-induced errors can substantially reduce perceived momentum (Asai et al., 2026), while fear of failure and concerns regarding shame or evaluation may intensify adolescents’ reactions to unsuccessful performance (Gustafsson et al., 2017). Youth sports research also links autonomy-supportive coaching with need satisfaction and perceived competence (Conroy and Coatsworth, 2007; Coatsworth and Conroy, 2009). More recent youth basketball studies indicate that contextual pressure, performance feedback, and coach self-regulation can shape athletes’ perceived coaching climate and psychological experiences (Gutman et al., 2025; Morbée et al., 2025).

Despite these findings, research on momentum, post-error emotion, attentional control, efficacy beliefs, and coaching has largely developed separately. It remains unclear how one error in youth basketball may evolve into repeated errors, or how coach feedback may interrupt this process. Accordingly, this Mini Review integrates emotion regulation, attentional control, and efficacy beliefs to propose a conceptual account of negative psychological momentum formation and examines social evaluation and coach feedback as potential amplifying or resetting mechanisms.

A focused narrative search was conducted using Web of Science, Scopus, PubMed, PsycINFO, and SPORTDiscus from database inception to 31 July 2026, with the final search conducted on 31 July 2026, and it was supplemented by backward and forward citation tracking. Search strings were adapted across databases and primarily combined terms related to psychological momentum and basketball/youth sports with terms related to error or failure, emotion regulation, anxiety, attentional control, self-efficacy, fear of failure, social evaluation, and coach feedback. Peer-reviewed studies were prioritized according to their direct relevance to post-error processes and their population and sport specificity: evidence from youth basketball was considered first, followed by evidence from basketball more broadly, then from youth team sports, and finally from general sports psychology research when direct evidence was unavailable. Within these levels, recent empirical studies were prioritized, while seminal theoretical work was retained where necessary. Given the narrative scope of this Mini Review, no formal systematic risk-of-bias assessment or systematic study-selection protocol was applied. For interpretive clarity, evidence was considered direct only when the relevant post-error or cross-possession relationship was examined in youth basketball; youth basketball studies that addressed only individual component constructs or contextual factors, along with studies from other populations or sports, were treated as indirect support for the proposed pathway.

## 2 Formation mechanisms of negative psychological momentum in youth basketball

### 2.1 Post-error negative affect and its persistence

An isolated error does not necessarily produce negative psychological momentum; a more critical issue is whether the affective response to that error persists into the next possession. In a small field study of female basketball players, happiness predicted greater successful involvement, whereas anger and embarrassment predicted greater unsuccessful involvement, suggesting that moment-to-moment emotional states may covary with subsequent performance quality (Uphill et al., 2014). Laboratory evidence outside of basketball has shown that error feedback can increase appraisals of the probability of future failure and that this perceived probability and cost of failure predict anxiety, although downstream effects on attention and performance were not demonstrated in that experiment (Harris et al., 2023). Emotion regulation may therefore influence whether an adverse event is prolonged psychologically: among competitive athletes, cognitive reappraisal has been associated with more adaptive emotional and psychobiosocial experiences (Robazza et al., 2023). In basketball, where the interval between possessions is often brief, unresolved anger, embarrassment, or anxiety may carry over to the next decision before emotional recovery is complete. This possibility is theoretically plausible but has not yet been directly established in youth basketball.

### 2.2 Impaired attentional control and task-irrelevant attention

A second mechanism concerns the allocation of limited attentional resources after an error. Attentional Control Theory proposes that anxiety weakens goal-directed attentional control and increases the influence of stimulus-driven or threat-related processing (Eysenck et al., 2007). Consistent with this account, expert athletes performing under pressure have reported directing their attention more frequently toward worries than toward movement execution (Oudejans et al., 2011). Basketball-specific evidence is particularly relevant: experimentally induced anxiety was shown to shorten the quiet-eye period and reduce free-throw success, indicating impaired goal-directed visual control under pressure (Wilson et al., 2009). More broadly, efficient basketball decisions depend on selective visual search and anticipation, whereas additional working memory load reduces visual search accuracy and increases response time and gaze demands (Li et al., 2023; Nian et al., 2023). Perceptual-tactical training in young basketball players was also shown to improve tactical perception and the generation of tactical solutions, highlighting the perceptual and decisional demands that could plausibly be disrupted by error-related worry, although post-error processes were not examined directly (Díaz-Rodríguez and Pérez-Córdoba, 2024). Time pressure similarly reduces decision accuracy and alters visual search characteristics in college basketball players (Guo and Wang, 2025), while imposed time constraints have been associated with greater anxiety, poorer shooting performance, and less consistent shooting kinematics (Kostrna, 2022). These studies do not show that a previous error directly disrupts attention in youth players. Nevertheless, they support the hypothesis that post-error rumination or evaluation concerns could compete with the information needed to read defenders, detect help defense, identify passing options, and select an appropriate shot.

### 2.3 Efficacy-related beliefs and repeated errors

Errors may also alter how athletes judge their capacity to execute the next action. Efficacy beliefs in basketball are sensitive to immediate task conditions: in children aged 9–12 years, modifying basket height and ball size changed both shooting performance and self-efficacy, providing direct youth-basketball evidence that perceived capability responds to the performance environment (Chase et al., 1994). During actual basketball competitions, self-confidence was also associated with performance components involving substantial working memory demands (Parfitt and Pates, 1999). In a larger sample spanning high school, university, and professional basketball players, sport confidence was found to be positively related to perceived performance and showed mediating relationships with competitive anxiety variables (Chun et al., 2022). Basketball players also reported using goal-directed self-talk for cognitive and motivational self-regulation when clearly losing or competing in close games, indicating that attentional and confidence-related states are actively managed during adverse competitive situations (Latinjak et al., 2019). Taken together, these findings suggest that repeated failure may become particularly disruptive when an athlete shifts from a specific appraisal—“that pass was late”—to a broader efficacy judgment—“I cannot execute today.” Such a shift could promote excessive caution or compensatory risk-taking, although these behavioral pathways remain hypotheses that require direct testing in youth basketball.

### 2.4 A proposed integrative self-reinforcing cycle

Evidence from elite American football indicates that failure on the immediately preceding play modestly increases the likelihood of another failure, while large-scale tennis data similarly show that prior errors predict subsequent errors and that this relationship becomes stronger under greater competitive pressure (Harris et al., 2019, 2021). Because these studies involve adult elite athletes in other sports, they provide indirect evidence rather than confirmation of negative momentum in youth basketball. No study identified in the present review directly tested the complete sequence at the possession level in youth basketball, and the directional links composing the proposed cycle therefore require direct testing in this population.

The literature nevertheless supports an integrative hypothesis: an initial error may generate residual negative affect, shift attention toward self-evaluative or threat-related information, weaken efficacy beliefs, and thereby impair the next decision or execution; a subsequent error may then reinforce the same psychological states. Negative psychological momentum can therefore be conceptualized as a potentially self-maintaining error–emotion–attention–efficacy–error cycle, rather than as a sudden loss of technical skill. As illustrated in Figure 1, this cycle may be further amplified by the social-evaluative characteristics of youth sports and potentially interrupted by coach-mediated emotional, attentional, and efficacy resetting. Importantly, the complete sequence remains a conceptual integration rather than an empirically established causal model in youth basketball.

**Figure 1:**
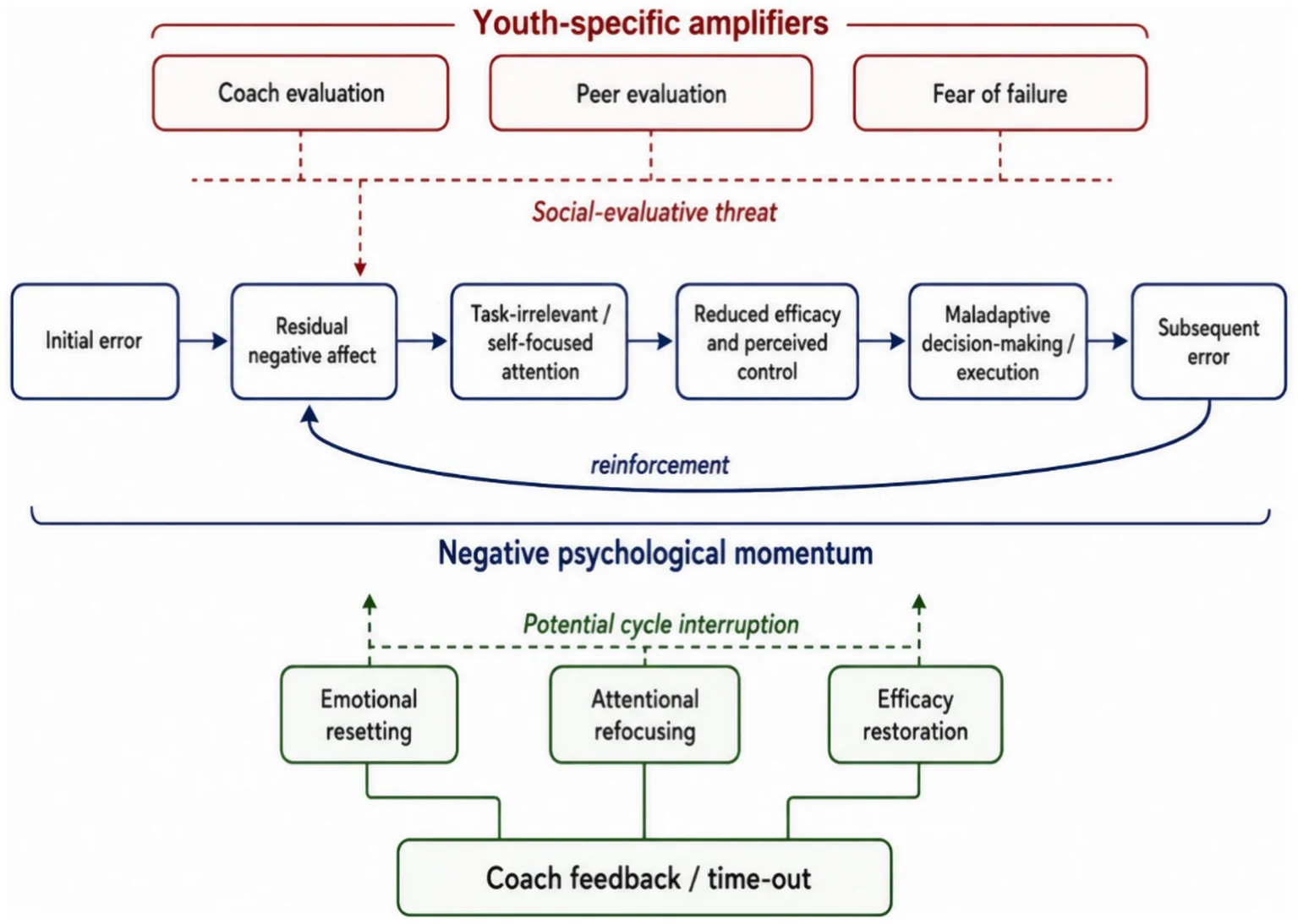
A proposed conceptual framework of negative psychological momentum formation and resetting in youth basketball. The arrows indicate proposed directional pathways rather than established causal relationships in youth basketball. Empirical support is stronger for individual component associations, whereas their post-error sequencing, social-evaluative amplification, and coach-mediated resetting remain hypotheses requiring direct possession-level testing.

## 3 Social-evaluative specificity of negative psychological momentum in youth basketball

### 3.1 Coach evaluation and social-evaluative threat

For young basketball players, an error occurs within an interpersonal hierarchy in which the coach controls playing time, role allocation, substitutions, and immediate feedback. A turnover or missed defensive assignment may therefore be interpreted as not only a task failure but also as information about how the athlete is being evaluated. In 1,166 athletes aged 9–18 years, including basketball players, perceived controlling coaching positively predicted somatic anxiety, worry, and concentration disruption, supporting the relevance of coach-created evaluative pressure (Ramis et al., 2017). Longitudinal evidence further showed that, among athletes aged 11–16 years, perceived coach autonomy support predicted a more task-involving peer climate and later intrinsic motivation (Jõesaar et al., 2012). More directly, among 681 basketball and handball players aged 14–17, a stronger task-involving coach climate was associated with greater satisfaction of basic psychological needs and lower fear of losing the interest of others or upsetting significant others (Valero-Valenzuela et al., 2024). Thus, the psychological impact of an error may partly depend on whether coach feedback frames it as correctable task information or as evidence of personal inadequacy.

### 3.2 Peer evaluation and the public nature of basketball errors

Basketball errors are highly visible: teammates immediately observe missed shots, turnovers, defensive breakdowns, and failed free throws. This public exposure may make failure relational rather than purely technical. Longitudinal research with British adolescent athletes showed that task-involving coach and peer climates predicted more adaptive emotional well-being than ego-involving climates, demonstrating that peers contribute to youth sports experience (Ntoumanis et al., 2012). Basketball-specific evidence is particularly relevant: across two studies of players aged 12–16 years, a task-involving peer climate positively predicted intrinsic motivation and negatively predicted fear of making mistakes (Moreno-Murcia and Conte Marín, 2011). Qualitative work with specializing athletes aged 9–18 similarly identified coaches and peers as salient social agents that shape the motivational meaning of sports experiences (Keegan et al., 2010). Although these studies do not directly measure post-error threat, they suggest that a mistake made in front of teammates may acquire psychological significance through perceived group reactions and standards.

### 3.3 Fear of failure and evaluation sensitivity as amplifiers

Not every young athlete should be expected to enter a negative momentum cycle after an error. Fear of failure may act as an individual-difference amplifier because failure can be appraised in terms of shame, reduced self-esteem, an uncertain future, or disappointing significant others. Interviews with young elite athletes revealed that these social and self-evaluative consequences are central reasons why failure is feared in sports (Sagar et al., 2007). For an evaluation-sensitive basketball player, a turnover may therefore shift attention from “what should I do next?” to “what does this mistake say about me?” or “what do my coach and teammates think of me?” This self-focused processing provides a plausible route through which social evaluation could intensify the emotion–attention–efficacy cycle described above. Youth specificity should therefore be understood not as an assumption that adolescents make more errors, but rather as the possibility that errors are more readily embedded in judgments about competence, belonging, and the approval of significant others.

## 4 Discussion

In this review, the proposed real-time resetting role of coach feedback is treated as a hypothesis rather than an established effect. Evidence showing that autonomy-supportive or controlling coaching climates shape the psychological experiences of youth athletes does not demonstrate that a brief coach response following an in-game error immediately alters recovery before the next possession. This broader coaching evidence is therefore treated as contextual support for the proposed resetting mechanisms rather than direct evidence of them. After an error, immediate feedback may influence whether negative affect, self-focused attention, and reduced efficacy persist into the next possession, which may be especially relevant in youth basketball because coaches are both tactical decision-makers and salient evaluators.

### 4.1 Emotional resetting: reducing carry-over from the previous error

A first potential function of coach feedback is to limit the emotional carry-over of an unsuccessful possession. A mastery-oriented coach intervention reduced the seasonal development of sport performance anxiety in young athletes relative to a control condition, suggesting that coach-created climates can alter how youth athletes experience competitive threats (Smith et al., 2007). In youth basketball, experimentally manipulated contextual pressure and performance feedback changed athletes’ perceptions of coach autonomy support and aspects of psychological need satisfaction, indicating that the coach’s evaluative context can reach athletes through interpersonal behavior (Morbée et al., 2025). Recent evidence from youth swimming further showed that coach education can increase athlete-reported empowering coaching behaviors and decrease disempowering ones, although in-game error recovery was not examined in this specific study (Moulds et al., 2026). Because these studies focus on broader coaching climates, contextual manipulations, or interventions rather than momentary post-error exchanges, they provide contextual support but do not directly demonstrate that brief supportive feedback after a turnover or missed assignment reduces residual threat before the next possession.

### 4.2 Attentional resetting: from outcome threat to executable cues

Following an error, commands such as “do not turn it over again” retain attention on the undesired outcome, whereas task-focused information can specify what should be detected or executed next. Research on youth ice hockey showed that feedback type and purpose, together with the match between preferred and perceived feedback, were related to athletes’ perceptions of the motivational climate (Stein et al., 2012). Although this does not establish an attentional effect, it suggests that feedback is interpreted contextually rather than received as neutral information. In basketball, timeouts can interrupt ongoing play, but their effects on subsequent momentum vary by team status and game context (Qiu et al., 2025). Thus, a timeout should not be treated as a psychological reset itself; its potential value may lie in creating a brief window to redirect attention from self-evaluation toward concrete cues, such as the first read after a pick-and-roll or the next defensive rotation. This pathway remains a mechanism to be tested.

### 4.3 Efficacy restoration: separating failed outcomes from global incompetence

Feedback may also prevent a specific error from becoming a broader judgment of personal ability. Positive competence feedback can enhance competence satisfaction and autonomous motivation, although direct performance benefits are not always observed (Mouratidis et al., 2008). Among female adolescent athletes, greater positive and informational coach feedback, combined with a stronger mastery climate, was associated with higher perceived competence, enjoyment, and intrinsic motivation (Weiss et al., 2009). For a young basketball player, “the read was correct; the pass was late” may therefore differ psychologically from “you cannot make that pass.” The former isolates a modifiable execution error while preserving information about effective judgment. Conceptually, this may restore perceived control and efficacy before the next possession and reduce the likelihood that one failed action becomes a generalized belief of incompetence. Direct youth basketball tests of this rapid efficacy-restoration process remain unavailable.

### 4.4 When feedback may amplify rather than reset negative momentum

Coach feedback should not be assumed to be protective. Across athlete samples, perceived coach control was associated more strongly with psychological need thwarting, whereas autonomy support was found to be more closely related to need satisfaction and positive functioning (Bartholomew et al., 2011). In youth soccer, autonomy-supportive coaching positively predicted basic psychological need satisfaction, whereas intimidation and the controlling use of rewards were associated with need frustration or lower need satisfaction (Sevil-Serrano et al., 2021). Punishment, humiliation, threats of substitution, or strongly outcome-controlling messages after an error may therefore intensify its social-evaluative meaning and sustain self-focused attention. Coach feedback may consequently act as either an interrupter or amplifier of the proposed error–emotion–attention–efficacy cycle.

The critical issue is not simply whether feedback is given, but whether it reduces emotional carryover, redirects attention to actionable information, and preserves competence and control. Accordingly, the feedback characteristics hypothesized to be most relevant to real-time post-error recovery are task-focused and actionable content, a calm and non-threatening emotional tone, delivery sufficiently close to the error to guide the next relevant action, and avoidance of person-focused criticism, humiliation, or threats. These characteristics should be regarded as candidate mechanisms for direct testing rather than established prescriptions for coaching practice. This integrated resetting account remains a conceptual hypothesis requiring possession-level testing in youth basketball (see Table 1).

**Table 1:** Evidence base and testable propositions for negative psychological momentum in youth basketball.

Proposed process	Current evidence base	Relevance to youth basketball	Testable proposition	Suggested measurement
Error → negative affect	Sport and basketball studies associate unsuccessful performance with negative affect; direct possession-level youth evidence remains limited (Uphill et al., 2014; Harris et al., 2023)	Errors occur during rapid transitions with little recovery time	Errors followed by greater negative affect predict poorer subsequent-possession performance	Event-coded errors + brief affect ratings
Negative affect → attentional disruption	Anxiety and attentional control research; basketball visual-attention evidence (Eysenck et al., 2007; Wilson et al., 2009; Oudejans et al., 2011)	Worry about previous plays or evaluations may compete with task-relevant cues	Post-error negative affect predicts greater self-focused attention and reduced task-relevant visual search	Eye tracking + state anxiety/affect
Attention → decision/execution impairment	Basketball studies support the roles of visual search, working memory load, and time pressure (Li et al., 2023; Nian et al., 2023; Díaz-Rodríguez and Pérez-Córdoba, 2024; Guo and Wang, 2025)	Rapid pick-and-rolls and defensive reads place high demands on selective attention	Greater post-error attentional disruption predicts slower or less accurate decisions	Video-based decision task / possession coding / gaze indices
Error → reduced efficacy	Evidence from basketball and other youth sports links perceived competence/confidence with performance context (Chase et al., 1994; Chun et al., 2022)	Young athletes may interpret public errors as information about their competence	Reduced post-error efficacy mediates the association between initial and subsequent errors	Momentary self-efficacy/confidence rating
Social evaluation as amplifier	Youth-sport evidence supports the roles of coach climate, peers, and fear of failure (Sagar et al., 2007; Ramis et al., 2017; Valero-Valenzuela et al., 2024)	Coach and peer reactions are immediately visible after basketball errors	Fear of failure and perceived evaluative threat strengthens the error–attention relationship	Fear-of-failure scale + perceived coach/peer evaluation
Coach feedback as reset	Research on youth coaching and feedback supports motivational and affective effects; direct evidence for negative psychological momentum is lacking (Smith et al., 2007; Weiss et al., 2009; Morbée et al., 2025)	Coaches can intervene between possessions or during timeouts	Supportive/task-focused feedback accelerates affective and attentional recovery relative to controlling feedback	Coded coach communication + repeated affect/attention measures
Full negative psychological momentum cycle	Individual links have converging evidence; the complete chain has not been directly tested (Den Hartigh et al., 2016; Harris et al., 2019, 2021)	Basketball permits possession-by-possession temporal analysis	Errors cluster when affect, attention, and efficacy disturbances persist across possessions	Multilevel/time-series or intensive longitudinal analysis

### 4.5 Conclusion and future directions

Negative psychological momentum in youth basketball may reflect a cross-possession psychological process rather than a sudden loss of technical ability. This review proposes that an initial error can leave a residual negative affect, disrupt task-relevant attention, and weaken efficacy-related beliefs, thereby potentially increasing the likelihood of maladaptive decisions and further errors. In youth settings, the public and evaluative nature of mistakes may intensify this cycle through coach and peer evaluation and fear of failure. Coach feedback may therefore represent an important contextual influence: supportive, task-focused communication may promote emotional, attentional, and efficacy resetting, whereas controlling feedback may prolong the disturbance. Because the complete error–emotion–attention–efficacy–error pathway has not been tested directly in youth basketball, future possession-level studies should simultaneously examine these processes and determine when coach feedback interrupts rather than amplifies negative momentum.
